# A single dose multi-ingredient pre-workout supplement enhances upper body resistance exercise performance

**DOI:** 10.3389/fnut.2024.1323408

**Published:** 2024-01-23

**Authors:** Kyle S. Beyer, Max Gadsden, Patrick Patterson-Zuber, Adam M. Gonzalez

**Affiliations:** ^1^Resistance Exercise, Physiology, and Sport Laboratory, Department of Health and Exercise Physiology, Ursinus College, Collegeville, PA, United States; ^2^Department of Allied Health and Kinesiology, Hofstra University, Hempstead, NY, United States

**Keywords:** dietary supplement, resistance training, reaction time, power, muscle endurance

## Abstract

**Introduction:**

Multi-ingredient pre-workout supplements (MIPS) are commonly used by individuals looking to enhance exercise performance and augment adaptations to training. However, the efficacy of commercially available MIPS is largely dependent on the ingredient profile, and new formulations should be investigated to determine their effectiveness. Therefore, the purpose of this study was to examine the effects of a commercially available MIPS product on performance during an upper body resistance exercise protocol.

**Methods:**

Twenty resistance-trained participants (10 men, 10 women) volunteered to complete this double-blind, placebo-controlled, crossover study consisting of 3 visits. Visit 1 consisted of body composition, 1-repetition maximum (1RM) testing, and familiarization. Visits 2 and 3 consisted of supplementation with either MIPS or placebo (PLA) 1 h prior to completion of an upper body resistance exercise workout during which power output, repetitions completed, rating of perceived exertion (RPE), and perceived recovery were recorded. Assessments of reaction time, isometric mid-thigh pull, and perceived levels of focus, energy, fatigue, and “muscle pump” were also completed before supplementation, 1 h after supplementation, and immediately after exercise.

**Results:**

Statistical analysis revealed significant main effects of trial for reaction time (*p* < 0.001) and bench press peak power (*p* = 0.026) indicating better performance during the MIPS trial. Furthermore, total number of repetitions completed significantly increased (*p* = 0.003) during the MIPS (96.90 ± 21.31 repetitions) trial compared to PLA (89.50 ± 18.37 repetitions). Additionally, overall session RPE was significantly lower (*p* = 0.002) during the MIPS (7.6 ± 1.2) trial compared to PLA (8.3 ± 0.9).

**Discussion:**

These findings suggest that acute supplementation with this MIPS improved upper body resistance exercise performance while reducing participant RPE. Further research should investigate the efficacy of chronic supplementation with this MIPS as the acute response provided an ergogenic benefit.

## 1 Introduction

Multi-ingredient pre-workout supplements (MIPS) have become increasingly popular among athletes and fitness enthusiasts in recent years (1). These supplements are designed to enhance athletic performance by providing a range of ingredients that are thought to improve energy, focus, and endurance (2). Previous research has shown that some ingredients commonly found in MIPS, such as caffeine and creatine, can improve muscular force, power, and endurance (3, 4). Other ingredients, such as beta-alanine and L-citrulline, have been shown to improve muscular endurance and delay fatigue (5, 6). While there is evidence to suggest that MIPS can be effective for enhancing acute exercise performance (2), there is also a great deal of variability in the ingredients and dosages used in these products (7). Therefore, the results of previous literature on MIPS cannot be extrapolated to all MIPS products available on the market.

A recently developed MIPS (SHIFTED Maximum Formula Pre Workout, SHIFTED LLC, Monteagle, TN, United States) contains a range of ingredients, including caffeine, beta-alanine, creatine, L-citrulline, beet root extract, taurine, red spinach extract, and betaine anhydrous, that have individually been shown to improve athletic performance. Furthermore, the dosing of each ingredient within this product are similar to previously reported effective dosages (7), and the combination of these ingredients may produce a synergistic effect on exercise performance. Previous research on this MIPS has demonstrated positive effects on mood and reaction time following a single acute dose, without altering countermovement jump performance (8). However, no study has investigated this MIPS during a resistance exercise workout, where it may exert effects by improving performance, delaying fatigue, and decreasing perceived effort, ultimately increasing exercise volume.

The purpose of this study is to examine the effect of an acute single dose of SHIFTED MIPS on upper body resistance exercise performance. The primary variable, resistance exercise performance, will be assessed by repetitions completed and rating of perceived exertion (RPE) measures. Secondarily, this study will investigate the effects of this MIPS on reaction time, muscular power, and perceived levels of energy, vigor, fatigue and “muscle pump.” It is our hypothesis that when compared to placebo the MIPS will improve all measures, including resistance exercise performance, power, and reaction time.

## 2 Materials and methods

### 2.1 Participants

An *a priori* power analysis (GPower 3.1; α = 0.05, β = 0.8) was conducted using a large effect size (*f* = 0.46) based upon changes in total repetitions completed in previous work (9), indicating a minimum of 12 participants to detect statistical significance. Twenty resistance-trained college-aged men (*n* = 10) and women (*n* = 10) volunteered to participate in this study. Prior to participation, participants completed a physical activity readiness questionnaire and written informed consent. Inclusion criteria required participants to have 1 year of resistance training experience (minimum of 2 days per week of resistance training session with free weights), competent form with barbell bench press and barbell row as determined by a Certified Strength and Conditioning Specialist, be free of any physical limitations or chronic illness that may affect performance and be free of any medications and performance enhancing drugs. All participants were caffeine habituated, reporting regular daily use of caffeine. This study was approved by the Ursinus College Institutional Review Board (KB-HEP-Mips-0322). Descriptive characteristics of participants are presented in Table 1.

**Table 1:** Participant descriptive data.

Sex	Age (y)	Height (cm)	Mass (kg)	Body fat (%)	1RM (kg)	RT Experience (y)
Male	20.5±1.1	180.1±5.1	106.3±21.2	23.4±7.2	122.4±33.6	6.4±1.4
Female	19.4±1.2	165.4±11.1	72.4±18.8	27.2±5.4	46.5±16.4	3.4±1.4

All data presented as mean ± standard deviation. 1RM, estimated bench press 1-repetition maximum; RT, resistance training.

### 2.2 Study design

**Figure 1:**
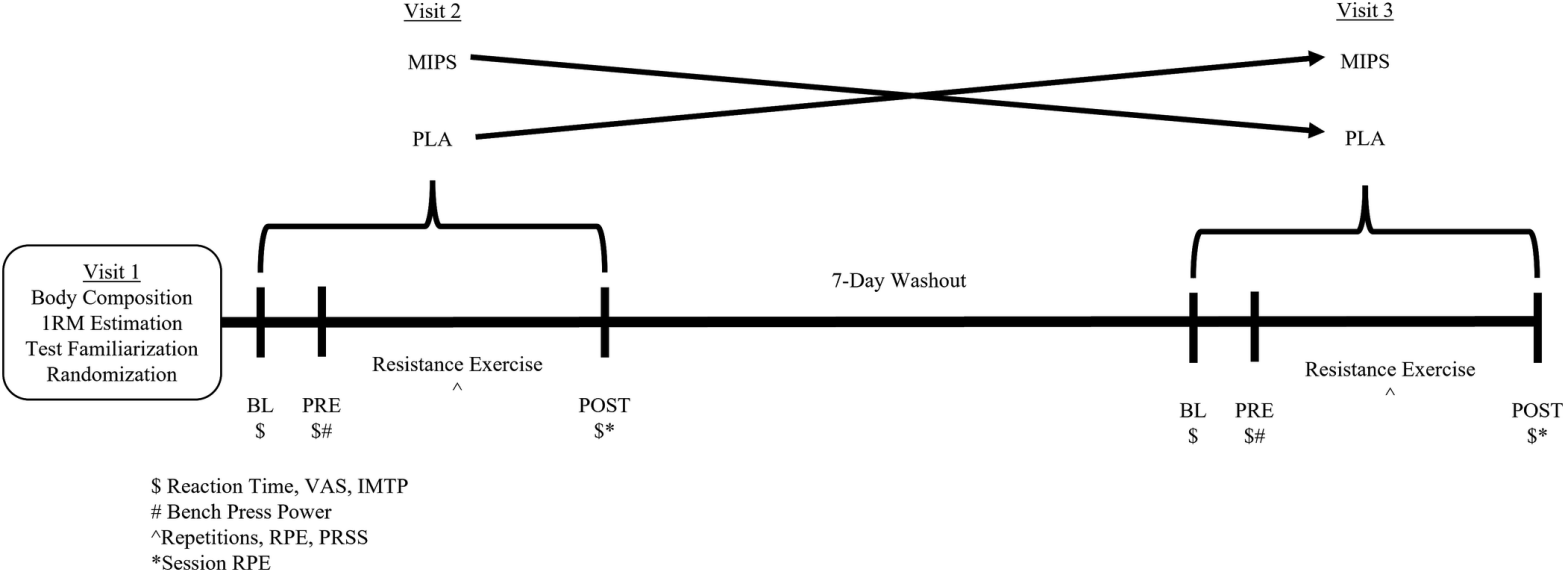
Study design. MIPS, multi-ingredient pre-workout supplement; PLA, placebo; BL, baseline; PRE, pre-exercise; POST, post-exercise; 1RM, 1-repetition maximum; VAS, visual analog scale; IMTP, isometric mid-thigh pull; RPE, rating of perceived exertion; PRSS, perceived recovery status scale.

This study utilized a double-blind, placebo-controlled, counter-balanced, crossover design study. All participants arrived at the Human Performance Laboratory on three separate occasions. The study design is presented in Figure 1. The first visit consisted of assessment of body composition, 1-repetition maximum (1RM) estimation, and familiarization with all testing procedures to be performed during the second and third visit. During the second and third visit, participants consumed either the MIPS or placebo (PLA) 1 h prior to completing an upper body resistance exercise workout during which repetitions completion, RPE, perceived recovery, and power (average and peak) were recorded. Assessments of reaction time, isometric mid-thigh pull (IMTP), and perceived levels of focus, energy, fatigue, and “muscle pump” were also completed before supplementation (BL), 1 h after supplementation (PRE), and immediately after exercise (POST). The upper body resistance exercise workout consisted of a bench press power protocol (2 sets of 2 repetitions at 75% 1RM with maximum velocity intent) followed by a strength-endurance bench press and bent over row protocol [5 repetition-maximum (RM) sets for the bench press exercise followed by 5 RM sets for the bent over row exercise using 75% 1RM].

Participants were instructed to continue their normal sleep, dietary, and exercise patterns throughout the data collection period but to avoid strenuous exercise and alcohol for 24 h prior to familiarization and the experimental trials. Experimental trials were scheduled for the same time of day between 8:00 and 11:00 a.m. and were separated by a 7 days washout period. For each experimental trial, participants were asked to arrive at the laboratory following an overnight fast (except for water). Participants were asked to refrain from using any ergogenic dietary supplement, including but not limited to protein powder, caffeine, nitric-oxide precursors, pre-workout, and creatine, throughout the duration of the study. Furthermore, participants who reported habitual use of creatine or beta-alanine were asked to refrain from supplementation for 4 weeks prior to study participation. Participants were also asked to record their nutritional intake for the day prior to their first experimental trial and were then instructed to duplicate it as closely as possible for their subsequent experimental trial. In addition, participants were asked to refrain from consuming other dietary supplements throughout the duration of the study.

### 2.3 Procedures

#### 2.3.1 Body composition

Initially, participants were assessed for height and body mass using stadiometer and digital scale (seca, Chino, CA, United States), respectively. Then, participants were assessed for body composition using bioelectrical impedance spectroscopy (SFB7, Impedimed, Carlsbad, CA, United States). Participants laid supine for 3–5 min to allow for fluid shifts. Two single-tab electrodes were placed on the right side of the body, 5 cm apart on both the dorsal surface of the wrist and dorsal surface of the ankle, respectively. The device measured total body water and extracellular fluid based on Cole modelling with Hanai mixture theory, which was then used to calculate percent body fat.

#### 2.3.2 1-repetition maximum testing

Participants were estimated for their 1RM on the barbell bench press and barbell bent over row exercises. Participants completed a general and specific warm-up consisting of dynamic stretches of the upper extremity and back, wall push-ups, and submaximal sets of bench press and bent over row. Following this standardized warm-up, participants completed a set to failure of bench press with a load of approximately 75–85% of the participants’ estimated 1RM. Based upon the number of repetitions performed and the weight used, the data was applied to a prediction formula (10) to estimate the 1RM. The same estimated 1RM procedure was then used for the bent over row exercise. A certified strength and conditioning specialist monitored and enforced proper technique.

#### 2.3.3 Supplementation

**Figure 2:**
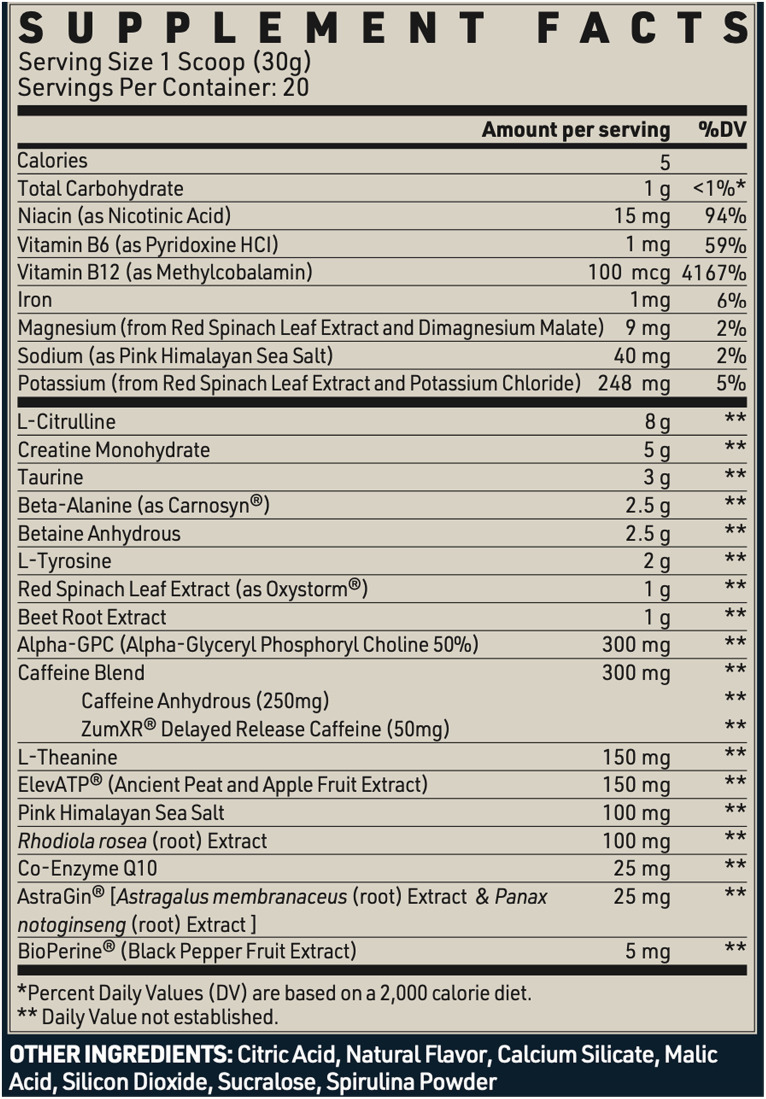
Supplement facts.

The MIPS utilized in this study is a commercially available product with a variety of common pre-workout ingredients (SHIFTED Maximum Formula Pre Workout, SHIFTED LLC, Monteagle, TN, United States). The supplement facts are presented in Figure 2. During the MIPS trial, participants consumed one serving of the supplement mixed with 12 fl. oz. of water. During the PLA trial, participants consumed the same volume of fluid flavored with a non-caloric placebo. The participants and test administrators were blinded to the supplement assignment, with a third party researcher mixing the assigned beverages. The MIPS and PLA were similar in taste, color, smell, and texture. Participants were provided 5 min to consume the entire beverage followed by 60 min to rest before beginning their PRE assessments.

#### 2.3.4 Reaction time

Reaction time was assessed with a visual-cognitive reactionary test (Blazepod, Play Coyotta Ltd., Tel Aviv, Israel) at BL, PRE, and POST. Six pods were affixed to the wall in a hexagon pattern, and the participant stood at a distance where each pod was within an arm’s reach. Pod placement and participant positioning was measured and replicated for all tests for each participant. During the 60 s trial, five of the six pods would illuminate at a time, with one pod illuminating blue and the other four illuminating green or red. Upon hitting the blue pod, all pods would turn off, then immediately re-illuminate with a different pattern. Participants were instructed to hit the blue pods as quickly as possible and ignore the red and green pods. Reaction time of each “hit” was recorded and the average reaction time (ms) was recorded for each trial. Participants completed two trials per assessment with a 30 s rest period and the fastest of the two trials was used for analysis.

#### 2.3.5 Isometric mid-thigh pull

Prior to completing physical assessments during the experimental trials, participants completed a general and specific warm-up consisting of dynamic stretches of the upper extremity and back, wall push-ups, and submaximal sets of bench press and bent over row. Participants completed an IMTP on a carbon fiber force plate with an isometric strength testing rig (C-Force Performance Platform, Innervations, West Perth, Australia) at BL, PRE, and POST. Testing was completed in accordance with previously published literature (11). Participants were positioned at the second pull of the clean with optimal knee (125–145°) and hip (140–150°) angles. Bar height was measured and replicated for all tests for each participant. All participants used a double overhand grip and were secured to the bar using wrist straps for all tests. Participants were instructed to pull on the bar and push their feet into the ground as hard and as fast as possible following the “pull” command. Two trials were performed for each test, with the highest peak force of the two being used for analysis. If the two trials were not within 250 N, then a third trial was performed. Internal reliability testing revealed excellent reliability (ICC_3,1_: 0.988, SEM: 86.04 N, MD: 238.49 N) for this IMTP testing protocol.

#### 2.3.6 Perceived levels of focus, energy, fatigue, and “muscle pump”

Participants rated their perceived levels of energy, focus, fatigue, and “muscle pump” using 15 cm visual analog scales at BL, PRE, and POST. “Muscle pump” was described to the participant as the feeling of temporary swelling of the muscle as a result of enhanced blood flow. All scales were framed as “My level of _______ is:” followed by a 15 cm line anchored with the words “Lowest” and “Highest.” Participants were instructed to place a vertical line at the spot that best described their current feeling. The validity and reliability of the visual analog scale were previously established (12).

#### 2.3.7 Upper body resistance exercise workout

The upper body resistance exercise workout consisted of a bench press power protocol followed by a strength-endurance protocol consisting of the barbell bench press and barbell bent over row exercises. The bench press power protocol was performed following a protocol consistent with previous literature (9). Participants completed 2 sets of 2 repetitions of bench press at 75% 1RM with intent to produce maximum velocity with 3 min of rest between sets. Peak power and average power of these repetitions were measured with a linear position transducer (Tendo Power Output Unit, Tendo Sports Machines, Trencin, Slovak Republic). This device was placed in a standardized location adjacent to the rack and with the cord attached to the lateral portion of the barbell collar. Power measures for each repetition were recorded and then averaged for each set.

Following a 5 min period of rest, participants completed 5 sets of barbell bench press to failure at 75% 1RM with 2 min of rest between sets. After a 2 min period of rest, participants completed 5 sets of barbell bent over row at 75% 1RM with 2 min of rest between sets. Failure was determined as the inability to complete a full range of motion repetition without assistance or with proper form. During each set, participants were encouraged to maintain a consistent cadence and not pause between repetitions. All experimental trials were overseen by the same certified strength and conditioning specialist.

#### 2.3.8 Perceived exertion and recovery

Prior to each bench press and bent over row set to failure, participants were asked to record their rating of recovery using the perceived recovery status scale (13). The perceived recovery status scale is a 0 to 10 scale with descriptive words used to assess the participant’s level of recovery. After the completion of each set to failure, participants were asked to record their RPE using the resistance exercise OMNI scale (14). At the end of the resistance exercise protocol, participants provided an overall RPE for the session using the OMNI scale. The OMNI scale is used for rating of perceived exertion with a 0 to 10 picture system corresponding to perceived intensity of physical exertion.

### 2.4 Statistical analysis

Reaction time, IMTP peak force, and perceived levels of focus, energy, fatigue, and “muscle pump” were analyzed with 2 × 3 (trial×time; MIPS vs. PLA × BL vs. PRE vs. POST) repeated measures analysis of variance. Repetitions completed, RPE, and perceived recovery for each set were assessed with 2 × 5 (trial×set: MIPS vs. PLA × Set 1 vs. Set2 vs. Set 3 vs. Set 4 vs. Set 5) repeated measures analysis of variance. Significant interactions and main effects were followed with Tukey-adjusted *post hoc* dependent *t*-tests. Bench press average and peak power, total repetitions completed, and session RPE were assessed with dependent *t*-tests. Alpha was set *a priori* to *p* ≤ 0.05. Cohen’s d effect sizes were calculated for all *t*-tests (15). All statistical procedures were conducted using JASP (Version 0.17.1). All data were reported as the mean ± SD.

## 3 Results

### 3.1 Reaction time

There was no significant trial×time interaction (*F* = 1.412, *p* = 0.256) for reaction time; however, there was a main effect of trial (*F* = 20.728, *p* < 0.001) and time (*F* = 36.958, *p* < 0.001). There was significantly faster reaction time during the MIPS trial (737.52 ± 123.27 ms) when compared to the PLA trial (793.30 ± 124.31 ms) regardless of time. Reaction time significantly (*p* < 0.001) improved from BL (800.53 ± 136.61 ms) to PRE (762.95 ± 132.91 ms) to POST (732.75 ± 114.57 ms) regardless of trial. Reaction time data are presented in Table 2.

**Table 2:** Participant reaction time, peak force, and perceived levels of focus, energy, fatigue, and “muscle pump” at baseline (BL), pre-exercise (PRE), and post-exercise (POST).

Variable	Treatment	BL	PRE		POST		Main effect of trial	Main effect of time	Interaction
Reaction time (ms)	PLA	826.1±130.5	798.7±131.4	#	755.2±112.8	†	<0.001	<0.001	0.256
	MIPS*	775.0±141.1	727.3±127.7		710.3±114.7				
IMTP peak force (*N*)	PLA	2645.1±1140.5	2699.3±1108.3		2516.1±941.6	†	0.351	0.001	0.128
	MIPS	2637.9±1140.7	2750.6±1161.9		2567.5±998.8				
Focus (AU)	PLA	7.73±2.35	8.54±1.89		9.11±2.63		0.235	0.067	0.855
	MIPS	8.46±2.42	9.39±2.66		9.58±2.64				
Energy (AU)	PLA	6.66±2.11	8.29±1.76		7.27±3.47	†	0.251	0.018	0.216
	MIPS	7.78±2.90	9.41±2.52		6.95±3.09				
Fatigue (AU)	PLA	6.58±3.04	5.53±2.83		8.98±3.88	†	0.189	<0.001	0.824
	MIPS	5.67±2.67	4.86±3.59		8.77±3.45				
Muscle pump (AU)	PLA	3.44±2.48	4.77±3.06		9.78±1.65	†	0.863	<0.001	0.422
	MIPS	3.98±2.79	4.53±3.29		9.66±2.59				

All data presented as mean ± standard deviation. AU, arbitrary units; IMTP, isometric mid-thigh pull; MIPS, multi-ingredient pre-workout supplement; ms, milliseconds; N, newtons; PLA, placebo; * Denotes significantly greater than PLA; # Denotes significant difference from BL; ^†^ Denotes significant difference from PRE.

### 3.2 IMTP peak force

There was no significant trial×time interaction (*F* = 2.191, *p* = 0.128) or main effect of trial (*F* = 0.922, *p* = 0.351) for IMTP peak force. There was a significant main effect of time (*F* = 8.504, *p* = 0.001), with significant decreases from PRE (2724.98 ± 1135.10 N) to POST (2541.82 ± 970.22 N) regardless of trial. IMTP peak force data are presented in Table 2.

### 3.3 Perceived levels of focus, energy, fatigue, and “muscle pump”

There were no significant trial×time interactions for levels of focus (*F* = 0.158, *p* = 0.855), energy (*F* = 1.594, *p* = 0.216), fatigue (*F* = 0.194, *p* = 0.824), or “muscle pump” (*F* = 0.882, *p* = 0.422). Further, there were no significant main effects of trial for any measure. However, there was significant main effects of time for energy (*F* = 4.469, *p* = 0.018), fatigue (*F* = 15.112, *p* < 0.001), and “muscle pump” (*F* = 89.162, *p* < 0.001), with significant changes from PRE (Energy: 8.85 ± 2.14 AU, Fatigue: 5.24 ± 3.21 AU, “Muscle Pump”: 4.65 ± 3.17 AU) to POST (Energy: 7.12 ± 3.28 AU, Fatigue: 8.87 ± 3.66 AU, “Muscle Pump”: 9.72 ± 2.12 AU). Perceived levels of focus, energy, fatigue, and “muscle pump” are presented in Table 2.

### 3.4 Bench press power

There was no significant difference in bench press average power (*t* = 1.879, *p* = 0.76, *d* = 0.420) between MIPS (428.55 ± 241.11 W) and PLA (399.65 ± 231.76 W) trials. However, there was a significantly greater (*t* = 2.416, *p* = 0.026, *d* = 0.540) bench press peak power after consuming MIPS (695.10 ± 457.25 W) when compared to PLA (614.65 ± 401.91 W).

### 3.5 Repetitions completed

**Figure 3:**
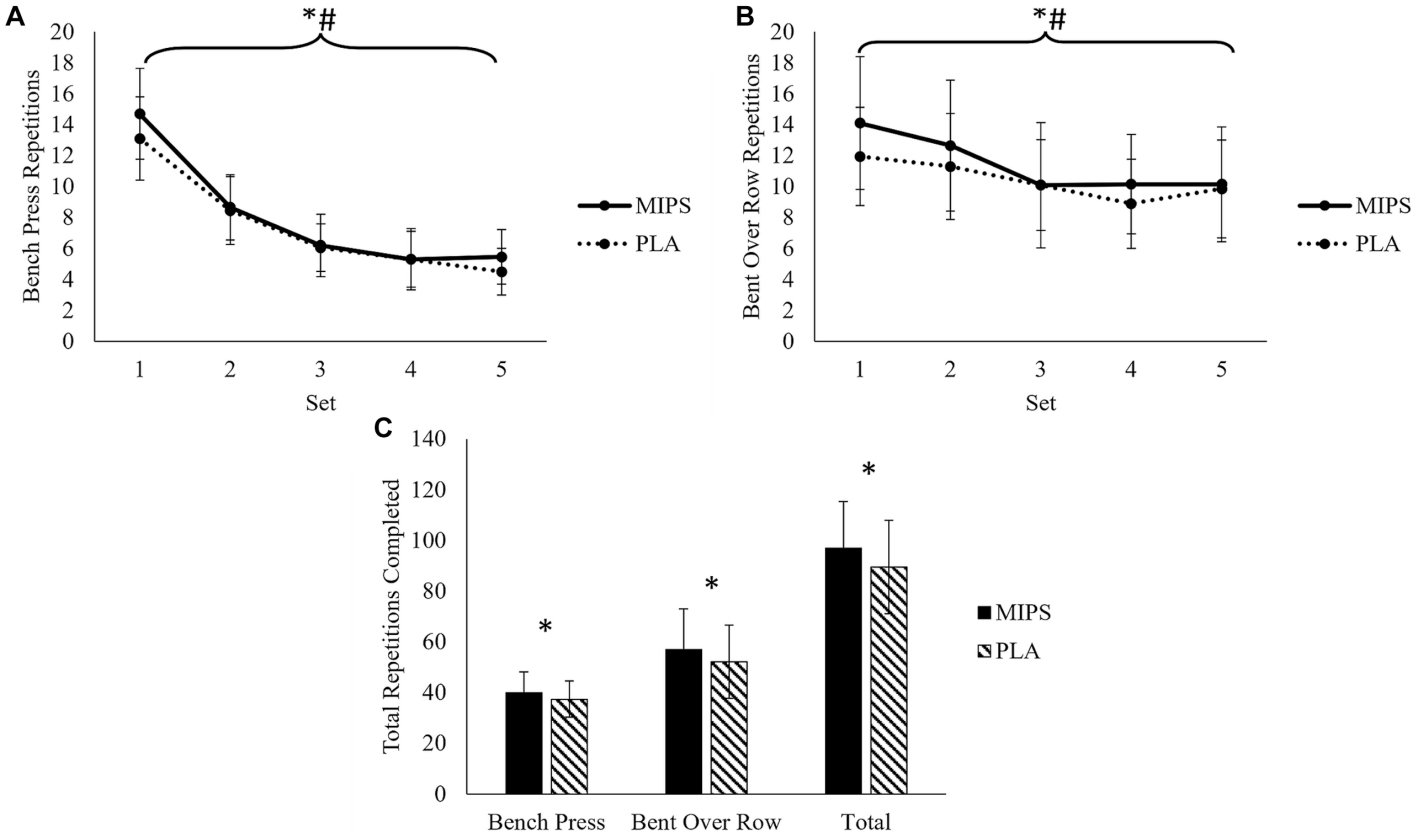
Repetitions completed during sets of bench press **(A)**, bent over row **(B)**, and total **(C)** during multi-ingredient pre-workout supplement (MIPS) and placebo (PLA) trials. * Denotes main effect of trial; ^#^ Denotes main effect of set.

There was no significant trial×set interaction for repetitions completed during bench press (*F* = 2.128, *p* = 0.085) or bent over row (*F* = 2.036, *p* = 0.098). However, there was a significant main effect of trial for both bench press (*F* = 9.931, *p* = 0.005) and bent over row (*F* = 7.461, *p* = 0.013), whereby more total repetitions were completed during MIPS trial for the bench press (MIPS: 39.95 ± 8.26 vs. PLA: 37.40 ± 7.18 repetitions) and bent over row (MIPS: 56.95 ± 16.01 vs. 52.10 ± 14.47 repetitions) compared to PLA. There were significant main effects of set for repetitions completed during bench press (*F* = 211.12, *p* < 0.001) and bent over rows (*F* = 21.18, *p* < 0.001). For bench press, there was a significantly (*p* < 0.001) reduced number of repetitions per set from set 1 (13.90 ± 2.90 repetitions) to set 2 (8.55 ± 2.12 repetitions) to set 3 (6.03 ± 1.66 repetitions) regardless of trial. For bent over rows, there was a significantly (*p* < 0.001) reduced number of repetitions per set from set 2 (11.95 ± 3.86 repetitions) to set 3 (10.03 ± 3.44 repetitions) regardless of trial. In terms of total repetitions completed per session, there were significantly (*t* = 3.403, *p* = 0.003, *d* = 0.761) more repetitions completed during the MIPS trial (96.90 ± 21.31 repetitions) compared to the PLA trial (89.50 ± 18.37 repetitions). Repetitions data are presented in Figure 3.

### 3.6 Perceived exertion and recovery

**Figure 4:**
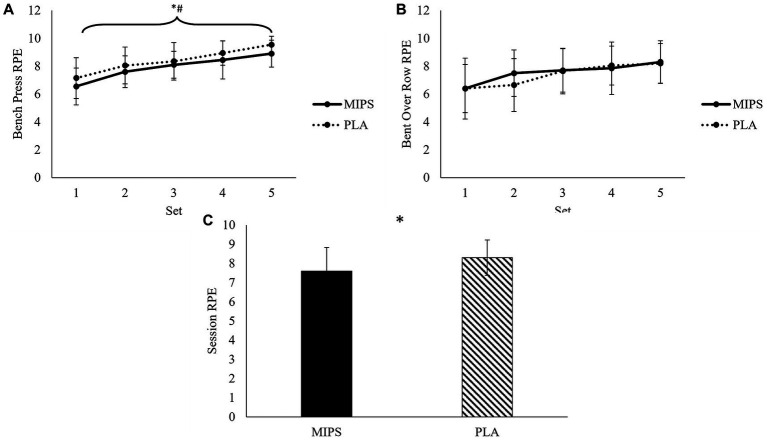
RPE after each set of bench press **(A)**, bent over row **(B)**, and overall session **(C)** during multi-ingredient pre-workout supplement (MIPS) and placebo (PLA) trials. * Denotes main effect of trial; ^#^ Denotes main effect of set.

There was a significant trial×set interaction for RPE during bent over rows (*F* = 2.918, *p* = 0.027), but *post hoc* tests revealed no significant differences between trials after any individual set. There was no trial×set interaction for RPE during bench press (*F* = 0.885, *p* = 0.477). However, significant main effects of trial (*F* = 10.05, *p* = 0.005) and set (*F* = 48.871, *p* < 0.001) were observed for RPE during bench press. *Post hoc* test revealed a significantly lower average RPE during the MIPS trial compared to the PLA during bench press. Furthermore, RPE significantly increased (*p* < 0.001) during bench press from set 1 (6.85 ± 1.41 AU) to set 2 (7.83 ± 1.24 AU), but no other significant increases were noted between subsequent sets. There were no significant trial×set interactions for perceived recovery status during bench press (*F* = 1.581, *p* = 0.188) or bent over row (*F* = 0.877, *p* = 0.482). For overall session RPE, a significantly lower (*t* = 3.61, *p* = 0.002, *d* = 0.810) RPE was noted during the MIPS trial (7.6 ± 1.2 AU) when compared to the PLA trial (8.3 ± 0.9 AU). RPE per set and overall sessions RPE data are presented in Figure 4.

## 4 Discussion

The effect of MIPS on exercise performance has been studied extensively (2), however it is difficult to draw comparisons between different ingredient formulations, and therefore, investigating new products brought to market has merit. The purpose of this study was to examine the acute effects of SHIFTED MIPS during an upper body resistance exercise workout. Our findings indicated that a single dose of this MIPS improved bench press peak power, total volume of training, and RPE.

While no significant trial×time interaction was detected for reaction time, overall significantly faster reaction times were noted during the MIPS trial when compared to the PLA trial. The MIPS investigated in this study has been previously shown to improve subjective measures and reaction time during a cognitively demanding task (8). In this study by Curtis et al. (8), a single serving of SHIFTED MIPS taken 45 min prior to a psychomotor vigilance test improved sustained attention and reaction time as well as levels of perceived fatigue and vigor. Other caffeine-containing MIPS formulations have also demonstrated similar improvements in reaction time following acute ingestion (16–19). As an adenosine antagonist and central nervous system stimulant, caffeine may be the ingredient to attribute these findings towards (20, 21), however other ingredients in the MIPS including L-tyrosine (22), alpha-glyceryl phosphoryl choline (alpha-GPC) (23), and *Rhodiola rosea* (24) have also shown to enhance cognitive properties. Additionally, a single dose of theanine has been shown to improve recognition visual reaction time compared to placebo (21) and work synergistically with caffeine (21, 25).

In the current study, acute supplementation with MIPS improved peak power during a bench press power protocol. This is in contrast to a previous study examining the effect of the same MIPS which reported no significant change in vertical jump performance when assessed 45 min following ingestion (8). However, other MIPS formulations have also shown no effect on vertical jump performance (26–28). The discrepancy in finding with the current study may be due to the different power assessment, with the current study using an upper body exercise while previous research used jumping tasks. For example, some studies have demonstrated that MIPS ingestion maintains upper body power after a fatiguing task but not lower body power (28, 29). Additionally, MIPS has shown to enhance peak and average power during bench press and squat exercises when averaged over an entire set or workout (30). Conversely, other research has demonstrated no effect of MIPS on upper body power output (31, 32). The disparity is likely attributed to the different MIPS formulations and the methodology by which power output was measured.

The current study demonstrated that MIPS may improve resistance to fatigue, allowing the participants to increase the number of repetitions performed over a series of bench press and bent over row sets. Along with improving total volume performed during the exercise protocol, MIPS also reduced the participants’ RPE. Previous studies investigating the effects of various MIPS products on resistance exercise performance have generally reported favorable results (27, 29, 30, 33); however, some studies have reported no effect of MIPS on exercise volume (31), or conflicting results between upper and lower body exercises (16, 26). In line with our current findings, a recent review of the literature on the effects of MIPS supported both enhancement in performance outcomes and self-reported levels of energy and fatigue (2). Additionally, as mentioned earlier, a previous investigation using SHIFTED MIPS reported improved self-reported feelings of fatigue and vigor following acute ingestion (8). Other studies investigating MIPS have also shown to improve ratings of perceived fatigue (16, 27); however, this finding is not always consistent (30, 34). This may be due largely to caffeine content as caffeine-containing MIPS have been shown to improve subjective feelings of energy but not caffeine-free MIPS (35).

The MIPS formula investigated in the current study produced a performance enhancing and anti-fatiguing effect as indicated by greater peak power expression and allowance for the completion of additional repetitions at a given workload. While it is not possible to fully elucidate the specific ingredients in the current MIPS responsible for the observed ergogenic effects, acute supplementation with various ingredients within the MIPS product have shown to exert similar performance enhancing effects. Specifically, caffeine has been shown to improve power production (36), total repetitions completed (37), and reduce RPE (38, 39). Acute beta-alanine supplementation has shown conflicting results with some research indicating no performance benefits during anaerobic exercise (40, 41), but a decreased RPE (41) and attenuated fatigue following exercise (42). Nitric oxide precursor ingredients such as L-citrulline and beetroot juice have been shown to acutely increase power and total resistance exercise repetitions completed (43). In addition, acute supplementation with red spinach extract has been shown to improve performance during a cycling time trial (44). Moreover, limited research on ElevATP™ has demonstrated improved exercise capacity following a single dose (45). In addition, acute supplementation with alpha-GPC may increase muscular force production during resistance exercise (46). Some of the included ingredients may have limited acute effects but exert larger benefits when consumed over a longer duration. Specifically, acute *Rhodiola rosea* supplementation has not been shown to improve reaction time (47), yet chronic supplementation for 4 weeks can improve reaction time (48). Chronic beta-alanine (49) and creatine monohydrate (50) supplementation have also been shown to increase resistance exercise performance, albeit these ingredients require loading periods of ~4 weeks to exert an ergogenic effect. In addition to their individual ergogenic effects, the ingredients within the MIPS may be working synergistically, amplifying the physiological effects and yielding greater performance benefits than when consumed alone.

The present investigation is not without limitations. All participants were regular caffeine users; therefore, the results of the study should not be applied to non-caffeine consuming individuals. Additionally, participants were recreationally trained individuals with at least 1 year of resistance training experience and various sporting backgrounds. It is unclear if the MIPS would produce similar benefits in untrained individuals or elite level athletes. While the resistance exercise protocol contained two different upper body exercise and stimulated physical and perceptual fatigued across ten sets to failure, no lower body exercises were included. Previous literature has reported discrepancies in the ergogenic benefits of a MIPS on upper and lower body exercise performance (26); therefore, this may be a future area of research for the current MIPS product. Lastly, the current study did not include any circulating markers to investigate the pharmacokinetics of the consumed MIPS or the mechanisms of the performance benefits; however, the presence of the improved reaction time, power, and performance seem to indicate that the MIPS is effective.

## 5 Conclusion

In conclusion, the current study provides evidence that acute supplementation with SHIFTED MIPS can enhance improve muscular endurance and power, and reduce perceived exertion during resistance training in resistance-trained men and women. Allowing an athlete to increase the amount of work performed over a series of sets may improve the anabolic stimulus and subsequent adaptation to strength training; however, acute improvements in performance do not necessarily translate to an enhanced long-term adaptation. Future research should investigate the chronic effects of this MIPS on training-induced gains in muscle strength and hypertrophy when accompanied by resistance training.

## Data availability statement

The raw data supporting the conclusions of this article will be made available by the authors, without undue reservation.

## Ethics statement

The studies involving humans were approved by Ursinus College Institutional Review Board. The studies were conducted in accordance with the local legislation and institutional requirements. The participants provided their written informed consent to participate in this study.

## Author contributions

KB: Conceptualization, Data curation, Funding acquisition, Investigation, Methodology, Supervision, Writing – original draft, Writing – review & editing. MG: Conceptualization, Data curation, Methodology, Writing – review & editing. PP-Z: Conceptualization, Data curation, Methodology, Writing – review & editing. AG: Conceptualization, Funding acquisition, Resources, Writing – review & editing.
